# Association between diabetes mellitus and multi-drug-resistant tuberculosis: evidence from a systematic review and meta-analysis

**DOI:** 10.1186/s13643-018-0828-0

**Published:** 2018-10-15

**Authors:** Balewgizie Sileshi Tegegne, Melkamu Merid Mengesha, Andreas A. Teferra, Mamaru Ayenew Awoke, Tesfa Dejenie Habtewold

**Affiliations:** 1 Department of Epidemiology and Biostatistics, College of Health and Medical Sciences, Haramaya University, Harar, Ethiopia; 2 Department of Epidemiology, University of Groningen, University Medical Center Groningen, Groningen, The Netherlands; 3 Division of Epidemiology, College of Public Health, The Ohio State University, Columbus, OH USA; 4 Amref Health Africa in Ethiopia, Monitoring, Evaluation and Research Unit, Addis Ababa, Ethiopia

**Keywords:** Diabetes mellitus, Tuberculosis, Multi-drug-resistant tuberculosis, Systematic review, Meta-analysis

## Abstract

**Background:**

Diabetes mellitus (DM) poses a significant risk for the development of active tuberculosis (TB) and complicates its treatment. However, there is inconclusive evidence on whether the TB-DM co-morbidity is associated with a higher risk of developing multi-drug-resistant tuberculosis (MDR-TB). The aim of this meta-analysis was to summarize available evidence on the association of DM and MDR-TB and to estimate a pooled effect measure.

**Methods:**

PubMed, Excerpta Medica Database (EMBASE), Web of Science, World Health Organization (WHO), and Global Health Library database were searched for all studies published in English until July 2018 and that reported the association of DM and MDR-TB among TB patients. To assess study quality, we used the Newcastle-Ottawa Scale for cohort and case-control studies and the Agency for Healthcare Research and Quality tool for cross-sectional studies. We checked the between-study heterogeneity using the Cochrane Q chi-squared statistic and *I*^2^ and examined a potential publication bias by visual inspection of the funnel plot and Egger’s regression test statistic. The random-effect model was fitted to estimate the summary effects, odds ratios (ORs), and 95% confidence interval (CIs) across studies.

**Results:**

This meta-analysis of 24 observational studies from 15 different countries revealed that DM has a significant association with MDR-TB (OR = 1.97, 95% CI = 1.58–2.45, *I*^2^ = 38.2%, *P* value for heterogeneity = 0.031). The significant positive association remained irrespective of country income level, type of DM, how TB or DM was diagnosed, and design of primary studies. A stronger association was noted in a pooled estimate of studies which adjusted for at least one confounding factor, OR = 2.43, 95% CI 1.90 to 3.12. There was no significant publication bias detected.

**Conclusions:**

The results suggest that DM can significantly increase the odds of developing MDR-TB. Consequently, a more robust TB treatment and follow-up might be necessary for patients with DM. Efforts to control DM can have a substantial beneficial effect on TB outcomes, particularly in the case of MDR-TB.

**Systematic review registration:**

PROSPERO CRD42016045692.

**Electronic supplementary material:**

The online version of this article (10.1186/s13643-018-0828-0) contains supplementary material, which is available to authorized users.

## Background

The global tuberculosis (TB) burden continues to be a major public health challenge despite efforts to reduce its impact. Globally in 2016, there were an estimated 10.4 million incident cases of TB, equivalent to 140 cases per 100, 000 population [1]. Of these incident cases of TB in 2016, an estimated 1.9 million were attributed to undernourishment, 1.0 million to HIV, 0.8 million to smoking, and 0.8 million to diabetes [1]. In the era of the sustainable development goals, post-2015, the “*End TB Strategy*” targets to reduce TB incidence by 80% by year 2030 [2]. However, the global epidemiological and demographic transitions pose significant challenge to TB control programs by changing the relative importance of different risk factors for TB [3, 4].

The global diabetes mellitus (DM) epidemic poses a significant bottleneck to the TB control program [3, 4]. The International Diabetes Federation (IDF) estimated that, globally in 2013, 382 million adults have diabetes of whom 80% live in low-and middle-income countries. Further increase in the global burden of diabetes is predicted, reaching 592 million by 2035 [5]. People with diabetes, compared to non-diabetic controls, were two- to three-fold more likely to develop TB [6, 7]. In 2013, an estimated 15% of adult cases of TB worldwide were attributed to diabetes, which corresponds to 1 million cases of diabetes-associated TB per year [3]. Impaired immunity in diabetic patients is thought to contribute to the evolution of latent TB infection to active cases. Moreover, people with TB who have DM have a poorer response to treatment than do those without DM, and are therefore at a higher risk of TB treatment failure, death, and relapse after cure [3, 8]. Treatment failure in turn adds another significant challenge to the global TB control program, a drug-resistant TB [1, 9].

Multi-drug-resistant tuberculosis (MDR-TB), resistance to at least isoniazid and rifampicin, results from either primary infection with resistant bacteria or may develop in the course of a patient’s treatment [9]. In 2016, there were an estimated 600,000 incident cases of MDR-TB. In the same year, an estimated 4.1% of new cases and 19% of previously treated TB cases had MDR-TB [1]. The emergence of multi-drug resistance across the world poses a global threat as the treatment is difficult, expensive, and a major healthcare cost burden to developing countries [10]. Most cases of MDR-TB arise from a mixture of physician error, inadequate and incomplete treatment, and patient non-compliance during treatment of susceptible TB [11, 12]. Research reports also indicate that patients with MDR-TB and a co-morbidity of DM have a poor treatment response compared with non-diabetic MDR-TB controls [13].

The additional risk of DM for the development of MDR-TB, however, remains controversial [14–16]. Many previous studies have found a 2.1 to 8.8 times increased risk of MDR-TB among TB patients co-morbid with diabetes [17–21]. In addition, observational studies from Israel, Georgia, and Mexico showed that TB patients with DM had a higher risk of developing MDR-TB [22–24]. In contrast, several others reported that there is no increased risk of MDR-TB among TB patients who have DM [25–28]. Similarly, none but one of the previously conducted systematic reviews and meta-analysis [29] reported DM as an independent risk factor for MDR-TB. However, the pooled estimate in that study was based on limited number of studies which mostly implemented a cross-sectional or case-control study design. By conducting a comprehensive search until July 2018, we identified more studies and included six new cohort studies [30–35]. Therefore, with the present systematic review and meta-analysis, we aimed to assess the pooled effect estimate of DM on the development of MDR-TB with the careful inclusion of data from appropriately conducted observational studies.

## Methods

### Registration

Our systematic review has been registered with the International Prospective Register of Systematic Reviews (PROSPERO) (http://www.crd.york.ac.uk/prospero/display_record.asp?ID=CRD42016045692). The protocol has been published elsewhere [36]. This review is written in accordance with the recommendations from the Preferred Reporting Items for Systematic Review and Meta-Analysis (PRISMA) statement guideline [37, 38], and a completed PRISMA checklist has been included (Additional file 1: Table S1).

### Eligibility criteria

We included all observational studies (cross-sectional cohort, case-control cohort, and prospective and retrospective cohorts) which reported the association of DM and MDR-TB among TB patients. All eligible studies published in English and prior to July 30, 2018, were included for the review.

### Data source and search strategy

PubMed, Excerpta Medica Database (EMBASE), Web of Science, and WHO Global Health Library databases were searched for all publications. We also searched cross-references of identified articles. In consultation with an experienced medical information specialist, a comprehensive search strategy has been developed (Table 1). Search results were compiled using citation management software (RefWorks 2.0; ProQuest LLC, Bethesda, MD, USA, http://www.refworks.com).

**Table 1:** Search strings used and number of identified abstracts per literature database

Component	PubMed	No. of hits	EMBASE	No. of hits	Web of Science	No. of hits	WHO Global Health Library	No. of hits
Diabetes mellitus	(“Diabetes Mellitus” [mesh] OR diabetes*[tiab] OR diabetic*[tiab] OR T2DM [tiab] OR T1DM [tiab] OR “T2 DM”[tiab] OR “T1 DM”[tiab])	612,633	(‘Diabetes Mellitus’/exp. OR (diabetes* OR diabetic* OR T2DM OR T1DM OR ‘T2 DM’ OR ‘T1 DM’): ab,ti)	1,006,444	TS = (diabetes* OR diabetic* OR T2DM OR T1DM OR “T2 DM” OR “T1 DM”)	649,798	((Diabetes Mellitus) OR diabetes* OR diabetic* OR (T2DM) OR (T1DM) OR (T2 DM) OR (T1 DM))	670,864
Multi-drug-resistant tuberculosis	(“Tuberculosis, Multidrug-Resistant”[Mesh] OR ((“Tuberculosis”[Mesh] OR tubercul*[tiab] OR tb[tiab] OR antitubercul*[tiab]) AND (“Drug Resistance, Multiple”[Mesh] OR multidrug resist*[tiab] OR multi-drug resist*[tiab] OR drug resist*[tiab] OR MDR [tiab] OR multiresist*[tiab] OR multi resist*[tiab])) OR rifampcin resist*[tiab] OR MDR-TB [tiab])	16,247	(‘Tuberculosis, Multidrug-Resistant’/exp. OR ((‘tuberculosis’/exp. OR (tubercul* OR tb OR antitubercul*): ab,ti) AND (‘multidrug resistance’/exp. OR (‘multidrug resist*’ OR ‘drug resist*’ OR MDR OR multiresist* OR ‘multi resist*’):ab,ti)) OR (‘rifampcin resist*’ OR MDRTB):ab,ti)	20,105	TS = (tubercul* OR tb OR antitubercul*) AND TS = (multidrug resist* OR drug resist* OR MDR OR multiresist* OR multi resist* OR rifampcin resist* OR MDRTB)	17,917	((Tuberculosis, Multidrug-Resistant) OR ((Tuberculosis) OR tubercul* OR tb OR antitubercul* AND (multidrug resist* OR multi-drug resist* OR drug resist* OR MDR OR multiresist* OR multi resist*) OR rifampcin resist* OR MDRTB))	52,110
Combined search	#1 AND #2	235*	#1 AND #2	525*	#1 AND #2	254*	#1 AND #2	768*

*Date of hits: July 30, 2018

### Study selection

Articles were screened and selected for full-text review if they met the following selection criteria: (1) provided or permitted the computation of an effect estimate of DM on the development of MDR-TB; (2) included TB patients (all type) and defined MDR-TB based on standard protocol; resistance at least to isoniazid and rifampicin [9]; and (3) defined DM based on any of the following: baseline diagnosis by self-report, medical records, laboratory test, or treatment with oral hypoglycemic medications or insulin. We excluded studies for any of the following reasons: citations without abstracts, anonymous reports, duplicate studies, case reports, or studies which did not compare MDR-TB among people with DM to people without DM, and systematic reviews and meta-analysis. Additionally, studies that either did not provide effect estimates in odds ratios, rate ratios, hazard ratios, and relative risks or did not allow for the computation of these values were excluded. Two authors (BS and MM) screened and checked full-text studies for inclusion independently. Any disagreement was resolved by discussion. If consensus could not be reached, a third author determined the eligibility and approved the final list of retained studies.

### Quality assessment and data extraction

Meta-analysis of observational studies present particular challenges because of potential biases in the original studies and differences in study designs that make the calculation of a single summary estimate of effect of exposure potentially misleading [39]. Thus, assessing quality of studies using a standardized tool helps to classify risk of bias which can help to explain variation in the results of included studies. Two authors (BS and MM) checked the quality of studies independently using Newcastle-Ottawa Scale (NOS) [40] for cohort and case-control studies and the Agency for Healthcare Research and Quality (AHRQ) [41] tool for cross-sectional studies as shown in Additional file 2: Table S2. Case-control and cohort studies qualified for inclusion if they scored 7 points or more from a total of 9 points in three domains of the equally weighted nine NOS components: selection (4 points), comparability (2 points), and exposure assessment (3 points). Cross-sectional studies were included in the analysis if they fulfilled all the four components (comparability, exposure, outcome measurement, and statistical analysis) of the AHRQ criteria. Structured data extraction form was constructed and pre-tested. For every study that met our eligibility criteria, two authors (BS and MM) independently extracted the title, name of authors, year of publication, country, study design, study population, sample size, data collection procedure, diagnosis of DM, and MDR-TB. Crude or adjusted effect sizes (ORs) with confidence intervals in the original studies were also extracted.

### Statistical analysis

We estimated pooled OR with their 95% CI to evaluate the association between DM and MDR-TB among TB patients. Potential sources of heterogeneity between the studies were examined by using the Cochrane Q chi-squared statistic and *I*^2^ [42, 43], where *I*^2^ > 75% suggested considerable heterogeneity. Presuming the variation of the true effect of DM on MDR-TB between studies, the random-effect model [44] was fitted to estimate the summary effect (ORs) and 95% CIs across studies. Subgroup analyses were performed by study type, variable adjustment, DM type, and TB type and by the income level of the country where the primary study was conducted. Publication bias was assessed with the funnel plot for asymmetry, Egger’s test, and Begger’s regression models [45]. To see the trend of evidence accumulation, we ran a cumulative meta-analysis. We conducted an influence analysis to observe the effect of omitting a single study on the overall pooled effect estimate [46]. All analyses were performed using STATA SE 14.2 (Stata Corporation, College Station, TX) [47]. All reported *P* values were two-sided at the level of 0.05.

## Results

### Search results

**Fig. 1:**
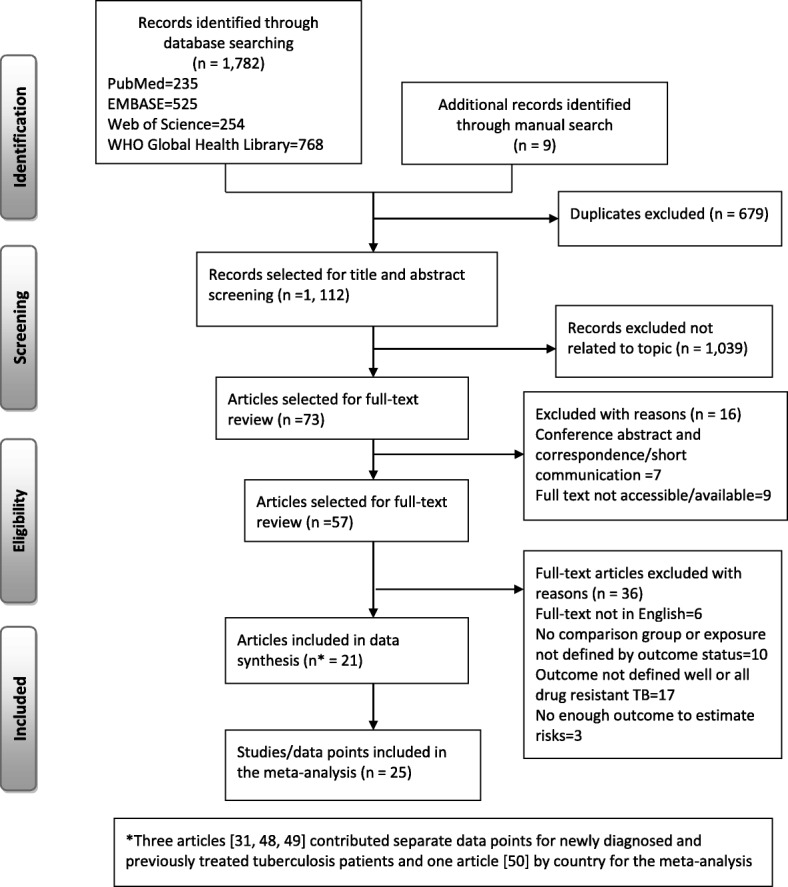
Flow chart showing study selection process and search results

We identified a total of 1782 studies based on the literature search in four databases including PubMed (235), EMBASE (525), Web of Science (254), and WHO Global Health library (768). Additionally, we found nine studies through a manual search (Fig. 1). After removal of duplicate studies, 1112 articles were screened based on titles and abstracts leaving 73 studies selected for a full-text review. Furthermore, 16 records were excluded from the full-text review (7 conference abstracts and correspondence/short communications and 9 full texts not accessible or available). Similarly, from the full-text review, we excluded 36 studies due to different reasons (6 were not written in English, 10 did not include comparisons, 17 did not define outcome clearly, and 3 had no enough outcome to estimate risks). List of the excluded articles after full-text review is available in Additional file 3. Finally, 21 articles were included for data synthesis. Additionally, three articles [31, 48, 49] reported separate effect sizes for newly diagnosed and previously treated TB patients, and one study [50] reported separate effect estimate by country (USA and Mexico), which resulted in a total of 25 studies or data points for analyses.

### Study characteristics

Table 2 presents the characteristics of all the 25 studies [14, 18–21, 23, 25, 28, 30–35, 48–54] with a total sample of 13,403 participants with TB. The studies were published between 2001 and 2018 and covered different geographical regions: 13 studies were in Asia (three in China, three in Taiwan, two in Georgia, one in Bangladesh, one in Indonesia, one in Iran, one in Thailand, and one in South Korea), three studies were in Europe (one in Spain, one in Portugal, and one in Turkey), eight studies were in the Americas (four in Mexico, two in the USA, and two in Peru), and one study was in Africa (Egypt). From the total studies included in the analysis, nine were case-control [18–21, 23, 25, 32, 52, 54], eight were cohort [14, 30, 31, 33–35, 53], and eight were cross-sectional [28, 48–51] by study design. No adjustment for confounding was done in 12 of the included studies, while the remaining studies adjusted for at least one confounding factor. The most commonly adjusted factors were age, sex, smoking status, and HIV status (Table 2). All the included studies passed quality assessment based on the Agency for Healthcare Research and Quality (AHRQ) [41] tool for cross-sectional studies and Newcastle-Ottawa Scale (NOS) [40] criteria for case-control and cohort studies (Additional file 2: Table S2).

**Table 2:** Characteristics of studies included in the meta-analysis assessing the association between diabetes mellitus and multi-drug-resistant tuberculosis

Author	County	Design	Sample size (TB-DM)	Data collection procedure	DM diagnosis	Diagnosis of TB	Diagnosis of MDR-TB	TB type	DM type	OR (95% CI)^††^	Adjustment to confounders
Baghaei P et al. [25]	Iran	CC	282 (24)	Record review	Not specified	Not specified	Not specified	All	Type not mentioned	0.68 (0.19–2.36)	No
Bashar M et al. [18]	USA	CC	155 (50)	Record review	Not specified	Not specified	Not specified	All	T1 and T2	5.30 (1.90–14.70)	Homelessness and HIV status
Carreira S et al. [34]	Portugal	RC	246 (123)	Record review	FBS/previous history of DM and receiving insulin or oral hypoglycemic agents	Not specified	Not specified	All	Type not mentioned	1.49 (0.24–9.06)	No
Chang JT [14]	Taiwan	PC	192 (60)	Record review/clinical/laboratory	FBS	Clinical/chest radiographs/sputum culture (LJ media)	DST (proportional methods)	New	T2	6.66 (0.68–65.38)	No
Fisher-Hoch SP et al. [50]	USA	CS	1442 (401)	Not mentioned	Self-report	Sputum Culture	DST	All	T2	2.14 (1.10–4.17)	Age, gender, alcohol and drug abuse, HIV infection, history of previous TB infection
Fisher-Hoch SP et al. [50]^†^	Mexico	CS	1436 (287)	Not mentioned	Self-report	AFB/culture	DST	All	T2	1.80 (1.13–2.87)	Age and gender
Gomez-Gomez A et al. [23]	Mexico	CC	175 (56)	Record review	FBS/HbA1c	Culture/PCR for mycobacterium TB complex	DST (proportion method)	All	T1 and T2	2.51 (1.11–5.67)	Age, sex, smoking history, chronic alcohol abuse, malnutrition, other illness conditions
Hafez S et al. [32]	Egypt	PC	40 (12)	Face-to-face interview/clinical/laboratory	FBS	AFB/culture (LJ)	DST (proportional method)	All	T1 and T2	2.96 (0.73–11.93)	No
Hsu A et al. [48]^*^	Taiwan	CS	139 (41)	Record review/laboratory	FBS	Culture	DST (proportional method)	Previously treated	T1 and T2	1.52 (0.59–3.95)	Age, sex
Hsu A et al. [48]	Taiwan	CS	869 (204)	Record review/lab	FBS	Culture	DST (proportional method)	New	T1 and T2	0.95 (0.34–2.68)	Age, sex
Jitmuang A et al. [54]	Thailand	CC	188 (31)	Record review	Not specified	Culture (LJ)	DST (proportional method)	All	Type not mentioned	1.28 (0.54–3.02)	No
Magee MJ et al. [31]^*^	Peru	PC	823 (143)	Record review/lab/interview	FBS/RBS/HbA1c/medication with Insulin or Oral hypoglycemic agents	AFB	DST (Griess method)	New	T1 and T2	0.45 (0.26–0.78)	No
Magee MJ et al. [53]	Georgia	PC	263 (37)	Face-to-face interview/clinical/lab	HbA1c¥	AFB/culture (LJ)/X-ray	DST (absolute concentration method)	New	Type not mentioned	2.27 (1.02–5.08)	Age, sex, HIV status and smoking
Magee MJ et al. [31]	Peru	PC	848 (43)	Record review/interview/lab	FBS/RBS/HbA1c/medication with Insulin or Oral hypoglycemic agents	AFB	DST (Griess method)	Previously treated	T1 and T2	1.10 (0.56–2.19)	No
Mi F et al. [49]^*^	China	CS	422 (144)	Record review	FBS	AFB/culture (LJ-media)	DST (proportional method)	New	T2	1.3 (0.6–2.8)	No
Mi F et al. [49]	China	CS	199 (43)	Record review	FBS	AFB/culture (LJ-media)	DST (proportional method)	Previously treated	T2	0.5 (0.2–1.1)	No
Min J et al. [52]	Korea, Rep.	CC	195 (55)	Record review	Record review	AFB/Chest radiographs/Culture	DST (LJ-media)	All	T1 and T2	2.68 (1.05–6.86)	Age and smoking
Perez-Navarro LM et al. [19]	Mexico	CC	409 (146)	Record review	FBS	AFB	NA	All	T2	3.50 (1.10–11.10)	Age
Perez-Navarro LM et al. [30]	Mexico	PC	507 (183)	Record review/self-report /lab	FBS	AFB	DST (BACTEC, MGIT)	New	T2	3.50 (1.60–7.10)	Age, sex, smoking, overcrowding
Saktiawati AMI et al. [35]	Indonesia	RC	356 (23)	Record review	FBS	AFB/positive chest X-ray/clinical	GeneXpert (MTB/RIF)/DST	All	T2		
Rifat M et al. [20]	Bangladesh	CC	1000 (83)	Face-to-face interview and record review	Not specified	AFB/X-ray	PCR (Xpert MTB/RIF)/culture/DST/Line probe assay	All	T2	2.56 (1.51–4.34)	Age, education, occupation and smoking status
Salindri A et al. [33]	Georgia	PC	268 (36)	Interview/lab	HbA1c	Molecular diagnostic test/AFB/culture (LJ)/clinical	DST (absolute concentration method)	New	Type not mentioned	2.51 (1.00–6.31)	Age, sex, education, income, smoking status, alcohol use, HIV status, kidney disease
Suarez-Garcia I et al. [21]	Spain	CC	696 (41)	Record review	Not specified	Culture	DST (agar proportion method)	All	Type not mentioned	1.84 (0.53–6.33)	No
Tanrikulu A et al. [28]	Turkey	CC	112 (9)	Record review	Not specified	Culture	DST (indirect proportion method)	All	Type not mentioned	4.65 (1.01–21.51)	No
Zhang Q et al. [51]	China	CS	2141 (203)	Record review	FBS	AFB/sputum culture	DST (proportional method)	All	T1 and T2	2.11 (1.42–3.11)	No

*T1* type I DM, *T2* type II, *DM* diabetes mellitus, *FBS* fasting blood sugar, *MDR-TB* multi-drug resistant tuberculosis, *TB* tuberculosis, *OR* odds ratio, *CI* confidence interval, *DST* drug susceptibility testing, *NA* not available, *FNAC* fine needle aspiration cytology, *PCR* polymerase chain reaction, *AFB* acid-fast bacilli, *PC* prospective cohort, *RC* retrospective cohort, *CC* case control, *CS* cross sectional, *LJ* Lowenstein Jensen, *HIV* human immunodeficiency virus, *TB-DM* refers to the number of TB patients co-morbid with DM in the sample

^*^These studies reported separate effect sizes for newly diagnosed and previously treated tuberculosis patients

^†^This study reported separate effect sizes by country (USA and Mexico)

^††^All effect sizes were presented as odd ratio

### Associations between DM and MDR-TB

We explored the influence of each individual study on the overall meta-analysis summary estimate. Table 3 shows the influence of omitting a single study on the overall summary estimate. Accordingly, we identified that omitting Magee MJ et al. [31] resulted in a large improvement on the overall summary estimate compared to the combined summary estimate obtained by omitting any one single study included in this meta-analysis (Table 3). This study was then excluded from the rest of the analysis resulting in 24 observational studies considered to conduct this meta-analysis.

**Table 3:** Single study influence analysis on the overall meta-analysis summary estimate of the association between diabetes mellitus and multi-drug-resistant tuberculosis

Study omitted	Effect size, OR	95% CI	
		LCL	UCL
Gomez-Gomez A et al. [23]	1.81	1.37	2.38
Magee MJ et al. [53]	1.82	1.38	2.40
Fisher-Hoch SP et al.^†^ [50]	1.84	1.38	2.45
Hsu A et al.* [48]	1.85	1.40	2.44
Saktiawati AMI et al. [35]	1.75	1.36	2.26
Rifat M et al. [20]	1.80	1.36	2.38
Bashar M et al. [18]	1.76	1.35	2.29
Perez-Navarro LM et al. [30]	1.77	1.35	2.33
Fisher-Hoch SP et al. [50]	1.82	1.38	2.41
Perez-Navarro LM et al. [19]	1.80	1.37	2.35
Salindri AD et al. [33]	1.81	1.38	2.38
Min J et al. [52]	1.81	1.37	2.38
Hsu A et al. [48]	1.88	1.43	2.46
Chang JT [14]	1.83	1.38	2.42
Zhang Q et al. [51]	1.82	1.37	2.43
Hafez S et al. [32]	1.81	1.38	2.38
Magee MJ et al.* [31]	1.88	1.43	2.48
Jitmuang A et al. [54]	1.86	1.41	2.46
Mi F et al.* [49]	1.93	1.49	2.49
Suarez-Garcia I et al. [21]	1.83	1.40	2.41
Magee MJ et al. [31]	1.97	1.58	2.45
Baghaei P et al. [25]	1.88	1.44	2.46
Tanrikulu A et al. [28]	1.79	1.37	2.35
Mi F et al. [49]	1.87	1.41	2.46
Carreira S et al. [34]	1.84	1.40	2.41
Combined	1.83	1.40	2.39

*UCL* upper confidence limit, *LCL* lower confidence limit, *CI* confidence interval, *OR* odds ratio

*These studies reported separate effect sizes for newly diagnosed and previously treated tuberculosis patients

^†^This study reported separate effect sizes by country (USA and Mexico)

**Fig. 2:**
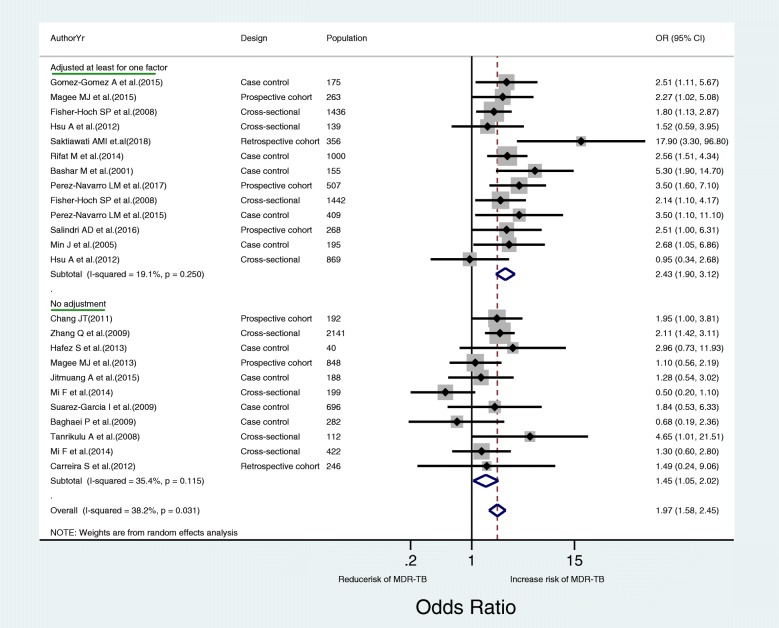
Forest plot showing the results of random effects meta-analysis of 24 observational studies. The horizontal line and vertical dotted line in the middle indicate the 95% confidence interval and its corresponding odds ratio (OR) estimate

Based on the meta-analysis of 24 observational studies, the overall pooled effect estimate was 1.97 (OR = 1.97, 95% CI 1.58 to 2.45, *I*^2^ = 38.2%, *P* value for heterogeneity = 0.031). This result indicated a 97% increased risk of MDR-TB among TB-DM co-morbid patients. The significant and positively increased risk of MDR-TB among TB-DM co-morbid patients remained in the same direction in a subgroup analysis by study characteristics. When seen by level of adjustment to a confounding factor, studies which adjusted for at least one confounding factor had more than a two-fold increased risk of MDR-TB (OR = 2.43, 95% CI 1.90 to 3.12) (Fig. 2). In a subgroup analysis of studies which adjusted for a minimum of one confounding factor, a strong positive association was observed by study design (cross-sectional, 4 studies, OR = 1.72, 95% CI 1.23 to 2.41; case-control, 5 studies, OR = 2.89, 95% CI 2.02 to 4.12; cohort, 4 studies, OR = 3.36, 95% CI 1.82 to 6.20) and method of TB diagnosis (culture confirmed, 8 studies, OR = 1.97, 95% CI 1.51 to 2.57; Sputum smear test, 5 studies, OR = 3.73 95% CI 2.33 to 5.97) (Table 4).

**Table 4:** Subgroup analyses of 24 observational studies on the association between diabetes mellitus and multi-drug-resistant-tuberculosis among tuberculosis patients co-morbid with diabetes mellitus

Study characteristics	Studies that adjusted for at least one covariate				Studies that did not adjustment for covariates			
	No. of studies	Pooled OR (95% CI)	*I*^2^ (%)	*P* value^¥^	No. of studies	Pooled OR (95% CI)	*I*^2^ (%)	*P* value^¥^
Study design								
Case control	5	2.89 (2.02, 4.12)	0.0	0.773	4	1.39 (0.79, 2.45)	0.0	0.459
Cohort	4	3.36 (1.82, 6.20)	40.2	0.171	3	1.47 (0.93, 2.34)	0.0	0.501
Cross sectional	4	1.72 (1.23, 2,41)	19.1	0.250	4	1.45 (0.68, 3.09)	73.2	0.011
Type of TB								
Both or not defined	7	2.72 (1.88, 3.94)	38.4	0.136	7	1.90 (1.40, 2.60)	0.0	0.492
New	5	2.36 (1.59, 3.51)	3.8	0.385	2	1.64 (0.99, 2.71)	0.0	0.436
Previously treated	1^†^				2	0.77 (0.36, 1.67)	35.4	0.115
Diagnosis of TB								
Culture confirmed	8	1.97 (1.51, 2.57)	0.0	0.831	8	1.60 (1.08, 2.38)	43.6	0.088
Sputum smear test only	5	3.73 (2.33, 5.97)	28.0	0.235	3	1.03 (0.58, 182)	0.0	0.736
Type of DM								
Type 2	6	2.67 (1.82, 3.93)	40.7	0.134	3	1.12 (0.52, 2.42)	67.3	0.047
Type 1 and type 2	5	2.22 (1.31, 3.76)	35.8	0.183	3	1.77 (1.08, 2.89)	35.5	0.212
Not defined	2	2.37 (1.29, 4.34)	0.0	0.872	5	1.46 (0.84, 2.51)	0.0	0.428
Diagnosis of DM								
HbA1c or FBS	3	2.42 (1.49, 3.93)	0.0	0.981	1^†^			
Only FBS	6	2.68 (1.45, 4.96)	53.5	0.056	6	1.50 (0.93, 2.42)	51.6	0.066
Self-report/unspecified	4	2.33 (1.65, 3.29)	21.5	0.282	4	1.49 (0.77, 2.89)	21.9	0.279
Country income level*								
Lower middle income	4	2.95 (1.69, 5.16)	40.6	0.168	1^†^			
Upper middle income	4	2.32 (1.65, 3.28)	0.0	0.418	7	1.27 (0.80, 2.01)	56.9	0.031
High income	5	2.13 (1.29, 3.52)	34.6	0.191	3	1.88 (1.07, 3.29)	0.0	0.963
Overall	13	2.43 (1.90, 3.12)	19.1	0.250	11	1.45 (1.05, 2.02)	35.4	0.115

*OR* odds ratio, *I*^*2*^ the variation in estimate attributable to heterogeneity, ¥ *P* value for heterogeneity, *CI* confidence interval, *TB* tuberculosis, *DM* diabetes mellitus, *FBS* fasting blood sugar, *HbA1c* glycosylated hemoglobin

^†^Effect estimate not pooled due limited number of available studies

*Income level based on World Bank Classification (*https://datahelpdesk.worldbank.org/knowledgebase/articles/906519-world-bank-country-and-lending-groups*)

**Fig. 3:**
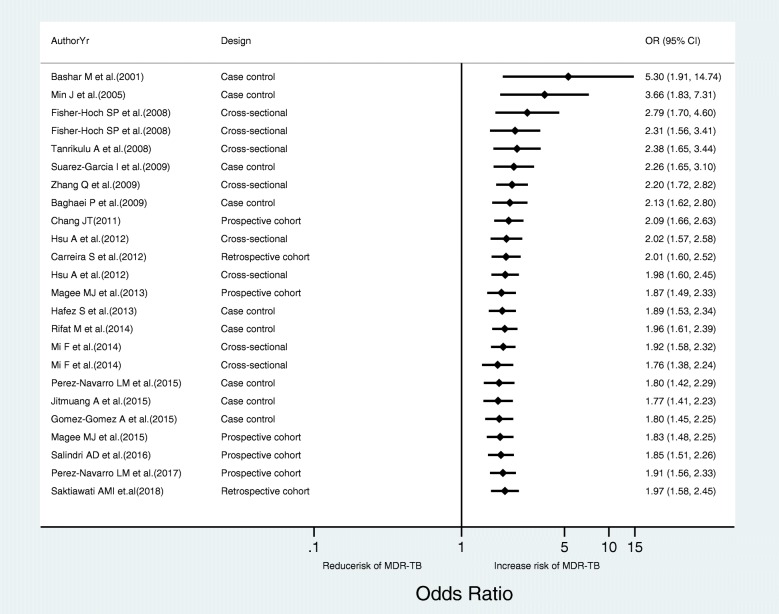
Cumulative forest plot showing the results of random effects meta-analysis for the 24 observational studies on the association of DM and MDR-TB. The first row shows the effect based on one study, the second row shows the cumulative effect based on two studies, and so on

To evaluate consistency of the evidence over the years along with its sufficiency, we ran a cumulative meta-analysis which calculates effect estimates as newer studies are added. Accordingly, between 2001 and 2014, there was a positive association between DM and MDR-TB but with a swinging effect size. However, with the addition of three case-control and four cohort studies between 2014 and 2018, a strong evidence that TB patients co-morbid with DM had an increased risk of developing MDR-TB has sustained (Fig. 3).

**Fig. 4:**
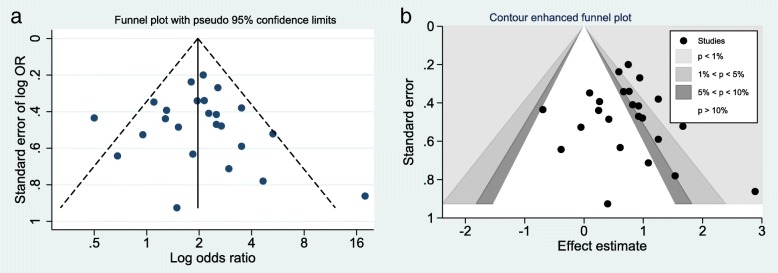
Funnel plot **a** and contour-enhanced funnel plot **b** of the included observational studies. In the contour-enhanced plot, the shaded region indicates areas of statistical significance, and non-statistical significance is represented in white. The vertical line corresponds to the summary log (OR) as estimated from the random-effect model (OR, odds ratio)

There was no significant publication bias found either by the Egger’s regression asymmetry test or by a funnel plot (Fig. 4a). The contour-enhanced funnel plot examination (Fig. 4b) confirms this, which distinguished between publication bias and other causes. It showed that small studies were found not only in the areas of statistical significance but also in areas of non-statistical significance.

## Discussion

In this study, we pooled estimates on the association between DM and MDR-TB from 24 observational studies and identified a significant and positive association with a low between-study heterogeneity. In a pooled estimate of studies which adjusted for at least one confounding factor, stronger association was observed by study design, type of TB, method of TB diagnosis, type of DM, method of DM diagnosis, and country income level.

The pooled effect estimate, in subgroup analyses of 13 studies which adjusted for at least one confounding factor, on the association between DM and MDR-TB (OR = 2.43, 95% CI 1.90 to 3.12, *I*^2^ = 19.1%, *P* value = 0.250) was consistent in the direction of association, but stronger than what was reported in a previous systematic review and meta-analysis (OR = 1.71, 95% CI 1.32 to 2.22, *I*^2^ = 46.8%, *P* value = 0.020) [29]. This difference could be due to the fact that Liu et al. [29] mixed three studies which reported unadjusted OR [21, 49, 54], while we included three new cohort studies that reported adjusted OR [30, 33, 35]. Compared to a similar prior meta-analysis [29], we included 10 additional studies [14, 25, 28, 30–35, 51] and supplemented this with strong analytical rigor. Thus, our meta-analysis fortifies the evidence base for the association between DM and MDR-TB. Our results are also in agreement with a review that showed TB and DM co-morbidity were more likely to be evident among people with anti-TB drug resistance [55], signifying a clear association between the two diseases. In another review, Baker et al. [8] did not find an increased risk of MDR-TB among people with DM. However, it is worth mentioning that this study aimed to review literature on the impact of DM on TB outcomes, and this particular sub-analysis included only four studies. Although not having MDR-TB as an outcome, another review noted significant associations in the pooled risk of association between DM and active TB without regarding the type of study design implemented [56].

Signifying a stability of association, we found a significant and positive association between DM and MDR-TB in subgroup analyses of studies which adjusted for at least one confounding factor. The association persisted irrespective of the country where the primary studies were conducted which covered diverse population groups, how DM or TB diagnosis was made, and study design of the primary studies. However, pooled estimate of a weaker strength was found in studies which did not adjust for at least one confounding factor (OR = 1.45, 95% CI 1.05 to 2.02). We included studies which reported unadjusted effect estimate to reduce inflation of the pooled effect estimate from the adjusted only analysis, increase the number of available studies for analysis, and widen the representativeness of our findings [57]. Although unadjusted findings lack adjustment by statistical methods, they attempted to adjust at the design level albeit still suffering from an unobserved confounding effect. Therefore, we suggest our findings should be interpreted with caution.

In subgroup analyses of studies which adjusted for at least one confounding factor on the association between DM and MDR-TB, there was an increased risk in lower middle-income countries (OR = 2.95, 95% CI 1.69 to 5.16) compared to that found in upper middle-income countries (OR = 2.32, 95% CI 1.65 to 3.28) and high-income countries (OR = 2.13, 95% CI 1.29 to 3.52). This might be due to more studies in high-income- or upper middle-income country settings diagnosed DM by blood test (FBS or HbA1c) or confirmed TB diagnosis based on culture which may have reduced bias due to misclassification [19, 23, 30, 48, 50, 52]. Consistent with our finding that there was an increased risk of MDR-TB among TB-DM co-morbid patients in lower middle-income countries, a meta-analysis by Al-Rifai et al. [56] reported that there was a higher risk of TB-DM association in lower middle-income countries compared to high-and upper middle-income countries.

A strong association was found in subgroup analyses by TB type in studies which reported adjusted estimate, where DM increased the risk of MDR-TB among new TB patients (OR = 2.36, 95% CI 1.59 to 3.51) and in those with undefined TB type (OR = 2.72, 95% CI 1.88 to 3.94). However, due to inadequate number of studies which adjusted for at least one confounding factor and included only previously treated TB patients, we did not present a pooled estimate on the risk of MDR-TB among previously treated TB-DM co-morbid patients. Therefore, we are not able to confirm whether DM is indeed not a risk factor for MDR-TB among previously treated TB patients. The meta-analysis by Liu et al. [29] also reported similar findings on the risk of any MDR-TB (OR = 1.94, 95% CI 1.42 to 2.65) and primary MDR-TB (OR = 1.69, 95% CI 1.09 to 2.62) among TB patients co-morbid with DM. Due to the addition of three cohort studies [30, 33, 35], which were not included in the Liu et al.’s meta-analysis [29], we reported a stronger pooled effect estimate for the increase in the risk of MDR-TB among new- and any-TB patients co-morbid with DM. Similarly, irrespective of the study design used in the primary studies which adjusted for at least one confounding factor, we consistently found a significant and positive association between DM and MDR-TB in cross-sectional (OR = 1.72 95% CI 1.23 to 2.41), case-control (OR = 2.89, 95% 2.02 to 4.12), and cohort studies (OR = 3.36, 95% CI 1.82, 6.20). Similar findings were reported by Liu et al. for case-control studies but not for cross-sectional studies [29]. Because only one cohort study was included [53] by Liu et al. [29], we cannot compare the pooled estimate from cohort studies in our meta-analysis to theirs. Though it is difficult to establish causality based on evidence from observational studies, we believe that these pooled estimates from studies which controlled for potential confounding factors further solidify the existence of a strong association between DM and MDR-TB among patients co-morbid with TB.

In this study, we have shown the most substantial evidence to date on the association between DM and MDR-TB by including a comprehensive list of studies carried out in various settings around the world. Methodologically, we conducted a cumulative meta-analysis to see the trend of evidence and performed a single study influence analysis and subgroup analysis. However, we would also like to acknowledge potential limitations of this study. Firstly, we have not included studies published in non-English languages. Given majority of MDR-TB cases and co-morbidities are from Russia, China, and India [1], exclusion of studies from these countries might bias our finding. Secondly, the inclusion of studies which did not consistently define the type of TB and did not control for potential confounding factors increased the observed heterogeneity among studies. However, in an attempt to deal with this, we have performed and reported sensitivity analysis. Finally, potential misclassification regarding a non-uniform diagnosis of DM and TB among participants included in the different studies might have contributed to varying risk estimates. This is particularly observed in the stratified analysis based on diagnosis of DM and TB, where higher estimates were observed for those diagnosed based on information obtained from medical records and self-reports, (diagnosis of DM: FBS from medical record, OR = 2.68, 95% CI 1.45 to 4.96; HbA1c, OR = 2.42, 95% CI 1.49 to 3.93; self-report, OR = 2.33,, 95% CI 1.65 to 3.29) and (diagnosis of TB: culture confirmed, OR = 1.97, 95% CI 1.51 o 2.57; AFB only from medical record, OR = 3.73, 95% CI 2.33 to 5.97).

In conclusion, our results demonstrate that a more robust TB treatment and follow-up might be necessary for patients with DM. In light of the global DM epidemic [5], this study emphasizes the message that there is a strong need for a bi-directional screening and co-management approach in the attempt to halt the TB-DM co-morbidity [58]. Even though countries representing diverse income levels were not included in our study, we stress the need to maximize efforts to prevent DM and TB co-morbidity and reduce the burden of MDR-TB in countries with varying economical standings. The integrated and collaborative effort between TB and DM control programs will ultimately count on achieving the global “*End TB Strategy*” [2]. Efforts to control DM can have a substantial beneficial effect on TB outcomes, particularly in the case of MDR-TB. Policymakers can focus on new targets pertaining to an enhanced care plan for DM patients with TB, particularly among the slightest evidence of problems with adherence or prolonged and complicated infections. Furthermore, focus on the early identification and treatment of individuals with the co-morbidity can result in an enhanced treatment outcome. We recommend future prospective cohort studies to focus on bacteriologically confirmed TB cases that objectively diagnose DM, with clearly defined types of both TB and DM coupled with robust controls for potential confounding.

## Additional files

Additional file 1: **Table S1.** Preferred Reporting Items for Systematic Reviews and Meta-analysis (PRISMA) 2009 checklist (DOCX 67 kb)

Additional file 2: **Table S2.** Study quality assessment results for case-control, cohort, and cross-sectional studies (DOCX 26 kb)

Additional file 3: List of studies excluded with reasons after full-text review (DOCX 20 kb)

## Abbreviations

AFB: Acid-fast bacilli
CI: Confidence interval
DM: Diabetes mellitus
DST: Drug susceptibility test
FBS: Fasting blood sugar
FNAC: Fine needle aspiration
HIV: Human immunodeficiency virus
LJ: Lowenstein Jensen
MDR-TB: Multi-drug-resistant tuberculosis
OR: Odds ratio
PCR: Polymerase chain reaction
TB: Tuberculosis
WHO: World Health Organization

## References

[1] World Health Organization (2017). Global tuberculosis report 2017.

[2] World Health Organization (2015). WHO end TB strategy: global strategy and targets for tuberculosis prevention, care and control after 2015.

[3] Lönnroth K, Roglic G, Harries AD (2014). Improving tuberculosis prevention and care through addressing the global diabetes epidemic: from evidence to policy and practice. Lancet Diabetes Endocrinol.

[4] Marais BJ, Lönnroth K, Lawn SD, Migliori GB, Mwaba P, Glaziou P (2013). Tuberculosis comorbidity with communicable and non-communicable diseases: integrating health services and control eff orts. Lancet Infect Dis 2013.

[5] IDF (2013). IDF Diabetes ATLAS.

[6] Alkabab YM, Al-Abdely HM, Heysell SK (2015). Diabetes-related tuberculosis in the Middle East: an urgent need for regional research. Int J Infect Dis.

[7] Jeon CY, Murray MB (2008). Diabetes mellitus increases the risk of active tuberculosis: a systematic review of 13 observational studies. PLoS Med.

[8] Baker MA, Harries AD, Jeon CY, Hart JE, Kapur A, Lönnroth K (2011). The impact of diabetes on tuberculosis treatment outcomes: a systematic review. BMC Med.

[9] World Health Organization (2010). World Health Organization multidrug and extensively drug-resistant TB (M/XDR-TB): 2010 global report on surveillance and response.

[10] Nations JA, Lazarus AA, Walsh TE (2006). Drug-resistant tuberculosis. Dis Mon.

[11] Jain A, Dixit P (2008). Multidrug resistant to extensively drug resistant tuberculosis: what is next?. J Biosci.

[12] Ormerod LP (2005). Multidrug-resistant tuberculosis (MDR-TB): epidemiology, prevention and treatment. Br Med Bull.

[13] Kang YA, Kim SY, Jo KW, Kim HJ, Park SK, Kim TH (2013). Impact of diabetes on treatment outcomes and long-term survival in multidrug-resistant tuberculosis. Respiration.

[14] Chang J, Dou H, Yen C, Wu Y, Huang R, Lin H (2011). Effect of type 2 diabetes mellitus on the clinical severity and treatment outcome in patients with pulmonary tuberculosis: a potential role in the emergence of multidrug-resistance. J Formosan Med Assoc.

[15] Kameda K, Kawabata S, Masuda N (1990). Follow-up study of short course chemotherapy of pulmonary tuberculosis complicated with diabetes mellitus. Kekkaku.

[16] Park S, Shin J, Kim J, Park I, Choi B, Choi J (2012). The effect of diabetic control status on the clinical features of pulmonary tuberculosis. Eur J Clin Microbiol Infect Dis.

[17] Baghaei P, Marjani M, Javanmard P, Tabarsi P, Masjedi MR (2013). Diabetes mellitus and tuberculosis facts and controversies. J Diabetes Metab Disord.

[18] Bashar M, Alcabes P, Rom WN, Condos R (2001). Increased incidence of multidrug-resistant tuberculosis in diabetic patients on the Bellevue Chest Service, 1987 to 1997. CHEST Journal.

[19] Pérez-Navarro LM, Fuentes-Domínguez FJ, Zenteno-Cuevas R (2015). Type 2 diabetes mellitus and its influence in the development of multidrug resistance tuberculosis in patients from southeastern Mexico. J Diabetes Complicat.

[20] Rifat M, Milton AH, Hall J, Oldmeadow C, Islam MA, Husain A (2014). Development of multidrug resistant tuberculosis in Bangladesh: a case-control study on risk factors. PLoS One.

[21] Suarez-Garcia I, Rodriguez-Blanco A, Vidal-Perez J, Garcia-Viejo M, Jaras-Hernandez M, Lopez O (2009). Risk factors for multidrug-resistant tuberculosis in a tuberculosis unit in Madrid, Spain. Eur J Clin Microbiol Infect Dis.

[22] Bendayan D, Hendler A, Polansky V, Weinberger M (2011). Outcome of hospitalized MDR-TB patients: Israel 2000–2005. Eur J Clin Microbiol Infect Dis.

[23] Gómez-Gómez A, Magaña-Aquino M, López-Meza S, Aranda-Álvarez M, Díaz-Ornelas DE, Hernández-Segura MG (2015). Diabetes and other risk factors for multi-drug resistant tuberculosis in a Mexican population with pulmonary tuberculosis: case control study. Arch Med Res.

[24] Kikvidze M, Mikiashvili L. Impact of diabetes mellitus on drug-resistant tuberculosis treatment outcomes in Georgia-cohort study. Eur Respir J. 2013;42.

[25] Baghaei P, Tabarsi P, Chitsaz E, Novin A, Alipanah N, Kazempour M (2009). Risk factors associated with multidrug-resistant tuberculosis. TANAFFOS-Journal of Respiratory Disease, Thoracic Surgery Intensive Care and Tuberculosis.

[26] Baghaei P, Tabarsi P, Abrishami Z, Mirsaeidi M, Faghani YA, Mansouri SD (2010). Comparison of pulmonary TB patients with and without diabetes mellitus type II. Tanaffos.

[27] Singla R, Khan N, Al-Sharif N, Al-Sayegh M, Shaikh M, Osman M (2006). Influence of diabetes on manifestations and treatment outcome of pulmonary TB patients. The International Journal of Tuberculosis and Lung Disease..

[28] Tanrikulu AC, Hosoglu S, Ozekinci T, Abakay A, Gurkan F (2008). Risk factors for drug resistant tuberculosis in southeast Turkey. Trop Dr.

[29] Liu Q, Li W, Xue M, Chen Y, Du X, Wang C (2017). Diabetes mellitus and the risk of multidrug resistant tuberculosis: a meta-analysis. Sci Rep.

[30] Perez-Navarro LM, Restrepo BI, Fuentes-Dominguez FJ, Duggirala R, Morales-Romero J, López-Alvarenga JC (2017). The effect size of type 2 diabetes mellitus on tuberculosis drug resistance and adverse treatment outcomes. Tuberculosis.

[31] Magee M, Bloss E, Shin S, Contreras C, Huaman HA, Ticona JC (2013). Clinical characteristics, drug resistance, and treatment outcomes among tuberculosis patients with diabetes in Peru. Int J Infect Dis.

[32] Hafez S, Elhefnawy A, Hatata E, El Ganady A, Ibrahiem M (2013). Detection of extensively drug resistant pulmonary tuberculosis. Egyptian Journal of Chest Diseases and Tuberculosis.

[33] Salindri AD, Kipiani M, Kempker RR, Gandhi NR, Darchia L, Tukvadze N (2016). Diabetes reduces the rate of sputum culture conversion in patients with newly diagnosed multidrug-resistant tuberculosis. Open Forum Infect Dis.

[34] Carreira S, Costeira J, Gomes C, André J, Diogo N (2012). Impact of diabetes on the presenting features of tuberculosis in hospitalized patients. Rev Port Pneumol (English Edition).

[35] Saktiawati AMI, Subronto YW (2018). Influence of diabetes mellitus on the development of multi-drug resistant-tuberculosis in Yogyakarta. Indones J Intern Med.

[36] Tegegne BS, Habtewold TD, Mengesha MM, Burgerhof JG (2017). Association between diabetes mellitus and multi-drug-resistant tuberculosis: a protocol for a systematic review and meta-analysis. Syst Rev.

[37] Beller EM, Glasziou PP, Altman DG, Hopewell S, Bastian H, Chalmers I (2013). PRISMA for abstracts: reporting systematic reviews in journal and conference abstracts. PLoS Med.

[38] Moher D, Schulz KF, Simera I, Altman DG (2010). Guidance for developers of health research reporting guidelines. PLoS Med.

[39] Stroup DF, Berlin JA, Morton SC, Olkin I, Williamson GD, Rennie D (2000). Meta-analysis of observational studies in epidemiology. A Proposal for Reporting JAMA.

[40] Wells G, Shea B, O’connell D, Peterson J, Welch V, Losos M (2000). The Newcastle-Ottawa Scale (NOS) for assessing the quality of nonrandomised studies in meta-analyses.

[41] Zeng X, Zhang Y, Kwong JS, Zhang C, Li S, Sun F (2015). The methodological quality assessment tools for preclinical and clinical studies, systematic review and meta-analysis, and clinical practice guideline: a systematic review. J Evid Based Med.

[42] Cochran WG (1954). The combination of estimates from different experiments. Biometrics.

[43] Higgins J, Thompson SG (2002). Quantifying heterogeneity in a meta-analysis. Stat Med.

[44] DerSimonian R, Laird N (1986). Meta-analysis in clinical trials. Control Clin Trials.

[45] Egger M, Davey Smith G, Schneider M, Minder C (1997). Bias in meta-analysis detected by a simple, graphical test. BMJ.

[46] Tob’ıas A. sbe26 (1999). Assessing the influence of a single study in the meta-analysis estimate. Stata Tech Bull.

[47] StataCorp (2015). Stata Statistical Software: Release 14.

[48] Hsu A, Lee J, Chiang C, Li Y, Chen L, Lin C (2013). Diabetes is associated with drug-resistant tuberculosis in Eastern Taiwan. Int J Tuberc Lung Dis..

[49] Mi F, Jiang G, Du J, Li L, Yue W, Harries AD (2014). Is resistance to anti-tuberculosis drugs associated with type 2 diabetes mellitus? A register review in Beijing, China. Glob Health Action.

[50] Fisher-Hoch SP, Whitney E, McCormick JB, Crespo G, Smith B, Rahbar MH (2008). Type 2 diabetes and multidrug-resistant tuberculosis. Scand J Infect Dis.

[51] Zhang Q, Xiao H, Sugawara I (2009). Tuberculosis complicated by diabetes mellitus at Shanghai Pulmonary Hospital. China Jpn J Infect Dis.

[52] Min Jinhong, Park Keeho, Whang Suhee, Kim Jinhee (2005). Risk Factors for Primary Multidrug Resistant Tuberculosis. Tuberculosis and Respiratory Diseases.

[53] Magee MJ, Kempker RR, Kipiani M, Gandhi NR, Darchia L, ea TN (2015). Diabetes mellitus is associated with cavities, smear grade, and multidrug-resistant tuberculosis in Georgia. Int J Tuberc Lung Dis.

[54] Jitmuang A, Munjit P, Foonglada S (2015). Prevalence and factors associated with multidrug-resistant tuberculosis at Siriraj Hospital, Bangkok, Thailand. Southeast Asian J Trop Med Public Health.

[55] Workneh MH, Bjune GA, Yimer SA (2017). Prevalence and associated factors of tuberculosis and diabetes mellitus comorbidity: a systematic review. PLoS One.

[56] Al-Rifai RH, Pearson F, Critchley JA, Abu-Raddad LJ (2017). Association between diabetes mellitus and active tuberculosis: a systematic review and meta-analysis. PLoS One.

[57] Voils CI, Crandell JL, Chang Y, Leeman J, Sandelowski M (2011). Combining adjusted and unadjusted findings in mixed research synthesis. J Eval Clin Pract.

[58] World Health Organization, and International Union against Tuberculosis and Lung Disease (2011). Collaborative framework for care and control of tuberculosis and diabetes.

