# Supplementary material for: Association between diabetes mellitus and multi-drug-resistant tuberculosis: evidence from a systematic review and meta-analysis
Source: Syst Rev. 2018 Oct 15;7:161. doi: 10.1186/s13643-018-0828-0 (PMC6190557; doi:10.1186/s13643-018-0828-0)
Supplement: Supplementary file 2 — Table S2. Study quality assessment results for case-control, cohort, and cross-sectional studies (DOCX 26 kb) [file 13643_2018_828_MOESM2_ESM.docx]

**Methodological quality assessment**

Table S2: Methodological quality assessment of case-control studies using Newcastle - Ottawa Scale (NOS)

| **First author, publication year** | **Criteria** | | | | | | | | |  |
| --- | --- | --- | --- | --- | --- | --- | --- | --- | --- | --- |
|  | **Selection** | | | | **Comparability** | | **Exposure** | | |  |
|  | **Adequacy of case definition** | **Representativeness of the cases** | **Selection of Controls** | **Definition of Controls** | **Study controls for most important factor(s)** | **Study controls for second important factor(s)** | **Ascertainment of exposure** | **Same method of ascertainment for cases and controls** | **Non-Response rate** | **Total score** |
| Min J et.al., 2005 | **1** | **0** | **0** | **1** | **1** | **1** | **1** | **1** | **1** | **7** |
| Rifat M et.al., 2014 | **1** | **1** | **1** | **1** | **1** | **1** | **1** | **1** | **1** | **9** |
| Jitmuang A et.al., 2015 | **1** | **1** | **1** | **1** | **0** | **0** | **1** | **1** | **1** | **7** |
| Gomez-Gomez A et.al., 2015 | **1** | **1** | **0** | **1** | **1** | **1** | **1** | **1** | **1** | **8** |
| Bashar M et.al., 2001 | **1** | **1** | **1** | **1** | **1** | **1** | **1** | **1** | **1** | **9** |
| Pérez-Navarro LM et al., 2015 | **1** | **1** | **1** | **1** | **1** | **1** | **1** | **1** | **1** | **9** |
| Baghaei p et.al., 2009 | **0** | **1** | **1** | **1** | **1** | **0** | **1** | **1** | **1** | **7** |
| Suàrez-García J, et.al, 2009 | **1** | **1** | **1** | **1** | **0** | **0** | **1** | **1** | **1** | **7** |
| Hafez S.A. et.al, 2013 | **1** | **1** | **1** | **1** | **0** | **0** | **1** | **1** | **1** | **7** |

*Note: each item account 1 point. (accept the study if total score ≥7)*

Table S2 Continued: Methodological quality assessment of cohort studies using Newcastle - Ottawa Scale (NOS)

| First author, publication year | Criteria | | | | | | | | |  |
| --- | --- | --- | --- | --- | --- | --- | --- | --- | --- | --- |
|  | **Selection** | | | | **Comparability** | | **Exposure** | | |  |
|  | **Adequacy of case definition** | **Representativeness of the cases** | **Selection of Controls** | **Definition of Controls** | **Study controls for most important factor(s)** | **Study controls for second important factor(s)** | **Ascertainment of exposure** | **Same method of ascertainment for cases and controls** | **Non-Response rate** | **Total score** |
| Magee MJ et.al., 2015 | **1** | **1** | **1** | **1** | **1** | **1** | **1** | **1** | **1** | **9** |
| Magee MJ et.al., 2013 | **1** | **1** | **1** | **1** | **1** | **1** | **1** | **1** | **1** | **9** |
| Salindri AD, et.al, 2016 | **1** | **0** | **1** | **1** | **1** | **1** | **1** | **1** | **1** | **8** |
| Carreira S et.al., 2012 | **1** | **1** | **1** | **0** | **1** | **0** | **1** | **1** | **1** | **7** |
| Perez-Navarro L.M. et al, 2017 | **1** | **1** | **1** | **1** | **1** | **1** | **1** | **1** | **1** | **9** |
| Saktiawati AMI et.al, 2018 | 1 | 1 | 1 | 1 | 1 | 1 | 1 | 1 | 1 | **9** |

*Note: each item account 1 point.* *(accept the study if total score ≥7)*

Table S2 Continued: Methodological quality assessment of cross-sectional studies using Agency for Healthcare Research & Quality (ARHQ) assessment scale

| **First author, publication year** | **Criteria** | | | | |
| --- | --- | --- | --- | --- | --- |
|  | **Comparability^1^** | **Exposure^2^** | **Outcome measurement^3^** | **Statistical analysis^4^** | **Total score** |
| Mi F et al., 2014 | 1 | 1 | 1 | 1 | 4 |
| Tanrikulu AC., et.al, 2008 | 1 | 1 | 1 | 1 | 4 |
| Fisher-Hoch SP et a.l, 2008 | 1 | 1 | 1 | 1 | 4 |
| Hsu AH et.al., 2012 | 1 | 1 | 1 | 1 | 4 |
| Zhang Q et al., 2009 | 1 | 1 | 1 | 1 | 4 |
| Chang JT et al., 2011 | 1 | 1 | 1 | 1 | 4 |

*Note: Accept the study if all criteria fulfilled*

^1^Comparability of cohorts on the basis of the design or analysis.

^2^Ascertainment of exposure based on validated measurement tool or non-validated measurement tool, but the tool is available or described OR No description of the measurement tool.

^3^Independent blind assessment, record linkage, self-report or no description.

^4^The statistical test used to analyse the data is clearly described and appropriate, and the measurement of the association is presented, including confidence intervals and the probability level (p value) or the statistical test is not appropriate, not described or incomplete.
