# Supplementary material for: Association between diabetes mellitus and multi-drug-resistant tuberculosis: evidence from a systematic review and meta-analysis
Source: Syst Rev. 2018 Oct 15;7:161. doi: 10.1186/s13643-018-0828-0 (PMC6190557; doi:10.1186/s13643-018-0828-0)
Supplement: Supplementary file 3 — List of studies excluded with reasons after full-text review (DOCX 20 kb) [file 13643_2018_828_MOESM3_ESM.docx]

**List of excluded studies after full text review**

**Non-English language articles**

1. Song QS, Cai CK, Ren YW, Lu XW. Risk factors for MDR-TB and XDR-TB in Dalian patients. Journal of Dalian Medical University. 2015; 57:45-8.
2. Yagi T, Yamagishi F, SASAKI Y, Hamaoka T, Kuroda F, Higurashi H. Clinical analysis of multidrug-resistant tuberculosis. Kekkaku (Tuberculosis). 2001;76(12):717-21.
3. Hiro Y. Pulmonary tuberculosis and diabetes mellitus. Joint study unit of national sanatorium (Japanese)
4. Fugino T, Hasewaga N, Satou R, Komatsu H, Kawada K. Attributable factors to the emergences of multidrug-resistance test results. Kekkaku. 1998;73(7):471-6.
5. Okumura M, Yoshiyama T, Ogata H, Morimoto K, Kokuto H, Kurashima A, Kudoh S. Factors related to the occurrence of multi-(extensively-) drug resistant tuberculosis (M/XDR-TB) in our hospital. Kekkaku:[Tuberculosis]. 2011 Nov;86(11):863-8.
6. Mendoza León C. Diabetes mellitus mal controlada como factor de riesgo para tuberculosis resistente en el Hospital Nacional Daniel Alcides Carrión durante el periodo 2010-2012.

**No comparison group or exposure not defined by outcome status**

1. Wang CS, Chen HC, Yang CJ, Tsai JR, Chong IW, Hwang JJ, Huang MS. Clinical characteristics of pulmonary tuberculosis patients from a southern Taiwan hospital-based survey. The Kaohsiung journal of medical sciences. 2008 Jan 1;24(1):17-24.
2. Datta BS, Hassan G, Kadri SM, Qureshi W, Kamili MA, Singh H, Manzoor A, Wani MA, u Din S, Thakur N. Multidrug-resistant and extensively drug resistant tuberculosis in Kashmir, India. The Journal of Infection in Developing Countries. 2009 Nov 21;4(01):019-23.
3. Raazi J, Prakash S, Parveen K, Shaikh S. Risk factors of multi-drug resistant tuberculosis in urban Allahabad, India. International Journal Of Community Medicine And Public Health. 2017 Jun 23;4(7):2383-8.
4. Li WB, Zhang YQ, Xing J, Ma ZY, Qu YH, Li XX. Factors associated with primary transmission of multidrug-resistant tuberculosis compared with healthy controls in Henan Province, China. Infectious diseases of poverty. 2015 Dec;4(1):14.
5. Kirenga BJ, Ssengooba W, Muwonge C, Nakiyingi L, Kyaligonza S, Kasozi S, Mugabe F, Boeree M, Joloba M, Okwera A. Tuberculosis risk factors among tuberculosis patients in Kampala, Uganda: implications for tuberculosis control. BMC public health. 2015 Dec;15(1):13.
6. Bates M, O’Grady J, Mwaba P, Chilukutu L, Mzyece J, Cheelo B, Chilufya M, Mukonda L, Mumba M, Tembo J, Chomba M. Evaluation of the burden of unsuspected pulmonary tuberculosis and co-morbidity with non-communicable diseases in sputum producing adult inpatients. PloS one. 2012 Jul 27;7(7):e40774.
7. Uchimura K, Ngamvithayapong-Yanai J, Kawatsu L, Ohkado A, Yoshiyama T, Shimouchi A, Ito K, Ishikawa N. Characteristics and treatment outcomes of tuberculosis cases by risk groups, Japan, 2007–2010. Western Pacific Surveillance and Response. 2013 Jan 29;4(1).
8. Shenjie T, Qing Z, Jinming Y, Yidian L, Wei S, Hua S, Lin F, Jin G, Xiaohui H, Lan Y, Heping X. Extensively drug-resistant tuberculosis at a tuberculosis specialist hospital in Shanghai, China: clinical characteristics and treatment outcomes. Scandinavian journal of infectious diseases. 2011 Apr 1;43(4):280-5.
9. Reis-Santos B, Gomes T, Locatelli R, de Oliveira ER, Sanchez MN, Horta BL, Riley LW, Maciel EL. Treatment outcomes in tuberculosis patients with diabetes: a polytomous analysis using Brazilian surveillance system. PLoS One. 2014 Jul 8;9(7): e100082.
10. Kikvidze M, Mikiashvili L. Impact of diabetes mellitus on drug-resistant tuberculosis treatment outcomes in Georgia-Cohort study.

**Outcome not defined well**

1. Baghaei P, Tabarsi P, Abrishami Z, MIRSAEDI M, Faghani YA, Mansouri SD, Masjedi MR. Comparison of pulmonary TB patients with and without diabetes mellitus type II.
2. Carrión Torres O, Cazorla Saravia P, Torres Sales JW, Yhuri Carreazo N, De La Cruz Armijo FE. Características del diagnóstico y tratamiento de la tuberculosis pulmonar en pacientes con y sin diabetes mellitus tipo 2. Rev Peru Med Exp Salud Publica. 2015 Dec; 32(4):680-6.
3. Nandakumar KV, Duraisamy K, Balakrishnan S, Sunilkumar M, Sagili KD, Satyanarayana S, Kumar A, Enarson DA. Outcome of tuberculosis treatment in patients with diabetes mellitus treated in the revised national tuberculosis control programme in Malappuram District, Kerala, India. PloS one. 2013 Oct. 14;8(10): e76275.
4. Dooley KE, Tang T, Golub JE, Dorman SE, Cronin W. Impact of diabetes mellitus on treatment outcomes of patients with active tuberculosis. The American journal of tropical medicine and hygiene. 2009 Apr 1;80(4):634-9.
5. Du J, Gao W, Ma Y, Zhong Q, Liang X, Fu Y, Ji B, Xie L, Huang X, Ge Q, Han X. Treatment effect analysis of the standard regimen and the optimized regimen for retreatment pulmonary tuberculosis complicated with diabetes. Zhonghua jie he he hu xi za zhi= Zhonghua jiehe he huxi zazhi= Chinese journal of tuberculosis and respiratory diseases. 2015 Dec;38(12):886-91.
6. Duangrithi D, Thanachartwet V, Desakorn V, Jitruckthai P, Phojanamongkolkij K, Rienthong S, Chuchottaworn C, Pitisuttithum P. Impact of diabetes mellitus on clinical parameters and treatment outcomes of newly diagnosed pulmonary tuberculosis patients in Thailand. International journal of clinical practice. 2013 ;67(11):1199-209.
7. Delgado-Sánchez G, García-García L, Castellanos-Joya M, Cruz-Hervert P, Ferreyra-Reyes L, Ferreira-Guerrero E, Hernández A, Ortega-Baeza VM, Montero-Campos R, Sulca JA, de Lourdes Martinez-Olivares M. Association of pulmonary tuberculosis and diabetes in Mexico: Analysis of the national tuberculosis registry 2000–2012. PloS one. 2015 Jun 15;10(6): e0129312.
8. Chiang CY, Bai KJ, Lin HH, Chien ST, Lee JJ, Enarson DA, Lee TI, Yu MC. The influence of diabetes, glycemic control, and diabetes-related comorbidities on pulmonary tuberculosis. PloS one. 2015 Mar 30;10(3): e0121698.
9. Alisjahbana B, Sahiratmadja E, Nelwan EJ, Purwa AM, Ahmad Y, Ottenhoff TH, Nelwan RH, Parwati I, Meer JW, Crevel RV. The effect of type 2 diabetes mellitus on the presentation and treatment response of pulmonary tuberculosis. Clinical Infectious Diseases. 2007 Aug 15;45(4):428-35.
10. Pérez-Navarro LM, Fuentes-Domínguez F, Morales-Romero J, Zenteno-Cuevas R. Factors associated to pulmonary tuberculosis in patients with diabetes mellitus from Veracruz, Mexico. Gaceta médica de México. 2011;147(3):219-25.
11. Delgado Rospigliosi JL, Seclen Santisteban SN, GOTUZZO HERENCIA E. Tuberculosis en pacientes con diabetes mellitus: Un estudio epidemiológico y clínico en el Hospital Nacional Cayetano Heredia. Revista Medica Herediana. 2006 Jul;17(3):132-40.
12. Gil-Santana L, Almeida-Junior JL, Oliveira CA, Hickson LS, Daltro C, Castro S, Kornfeld H, Netto EM, Andrade BB. Diabetes is associated with worse clinical presentation in tuberculosis patients from Brazil: a retrospective cohort study. PLoS One. 2016 Jan 11;11(1): e0146876.
13. Kikvidze M, Ikiashvili L. Comorbidities and MDR-TB treatment outcomes in Georgia-2009-11 cohort. European Respiratory Journal. 2014 Sep 1;44(Suppl 58):P1444.
14. Mehta S, Yu EA, Ahamed SF, Bonam W and Kenneth J. Rifampin resistance and diabetes mellitus in a cross-sectional study of adult patients in rural South India. BMC Infectious Diseases. 2015; 15:451. Doi: 10.1186/s12879-015-1204-5.

**Focused on all drug resistant tuberculosis**

1. Abdelbary BE, Garcia-Viveros M, Ramirez-Oropesa H, Rahbar MH, Restrepo BI. Tuberculosis-diabetes epidemiology in the border and non-border regions of Tamaulipas, Mexico. Tuberculosis. 2016 Dec 1;101: S124-34.
2. Gomes M, Correia A, Mendonça D, Duarte R. Risk factors for drug-resistant tuberculosis. Journal of Tuberculosis Research. 2014 Sep 3;2(03):111.
3. Fescina PM, Membriani E, Limongi L, Putruele A. Incidencia de la resistencia a drogas en tuberculosis y su asociación a comorbilidades en pacientes tratados en un hospital universitario. Revista americana de medicina respiratoria. 2013 Jun;13(2):64-70.

**No adequate cases to estimate association (OR, odds ratio)**

1. Singla R, Khan N, Al-Sharif N, Al-Sayegh MO, Shaikh MA, Osman MM. Influence of diabetes on manifestations and treatment outcome of pulmonary TB patients. The International Journal of Tuberculosis and Lung Disease. 2006 Jan 1;10(1):74-9.
2. Baghaei P, Tabarsi P, Moniri A, Marjani M, Velayati AA. Impact of diabetes mellitus on tuberculosis drug resistance in new cases of tuberculosis. International Journal of Mycobacteriology. 2015 Mar 1; 4:128.
3. Viswanathan V, Vigneswari A, Selvan K, Satyavani K, Rajeswari R, Kapur A. Effect of diabetes on treatment outcome of smear-positive pulmonary tuberculosis—a report from South India. Journal of Diabetes and its Complications. 2014 Mar 1;28(2):162-5.
